# Extent of Mitochondrial Hexokinase II Dissociation During Ischemia Correlates With Mitochondrial Cytochrome c Release, Reactive Oxygen Species Production, and Infarct Size on Reperfusion

**DOI:** 10.1161/JAHA.112.005645

**Published:** 2013-02-22

**Authors:** Philippe Pasdois, Joanne Elizabeth Parker, Andrew Philip Halestrap

**Affiliations:** 1 School of Biochemistry and The Bristol Heart Institute, University of Bristol, Bristol, UK (P.P., J.E.P., A.P.H.)

**Keywords:** hexokinase, ischemia/reperfusion injury, mitochondria, permeability transition pore, reactive oxygen species

## Abstract

**Background:**

The mechanisms by which ischemic preconditioning (IP) inhibits mitochondrial permeability transition pore opening and, hence, ischemia–reperfusion injury remain unclear. Here we investigate whether and how mitochondria‐bound hexokinase 2 (mtHK2) may exert part of the cardioprotective effects of IP.

**Methods and Results:**

Control and IP Langendorff‐perfused rat hearts were subject to ischemia and reperfusion with measurement of hemodynamic function and infarct size. Outer mitochondrial membrane (OMM) permeabilization after ischemia was determined by measuring rates of respiration and H_2_O_2_ production in the presence and absence of added cytochrome c in isolated mitochondria and permeabilized fibers. IP prevented OMM permeabilization during ischemia and reduced the loss of mtHK2, but not Bcl‐x_L_, observed in control ischemic hearts. By contrast, treatment of permeabilized fibers with glucose‐6‐phosphate at pH 6.3 induced mtHK2 loss without OMM permeabilization. However, metabolic pretreatments of the perfused heart chosen to modulate glucose‐6‐phosphate and intracellular pH_i_ revealed a strong inverse correlation between end‐ischemic mtHK2 content and infarct size after reperfusion. Loss of mtHK2 was also associated with reduced rates of creatine phosphate generation during the early phase of reperfusion. This could be mimicked in permeabilized fibers after mtHK2 dissociation.

**Conclusions:**

We propose that loss of mtHK2 during ischemia destabilizes mitochondrial contact sites, which, when accompanied by degradation of Bcl‐x_L_, induces OMM permeabilization and cytochrome c loss. This stimulates reactive oxygen species production and mitochondrial permeability transition pore opening on reperfusion, leading to infarction. Consequently, inhibition of mtHK2 loss during ischemia could be an important mechanism responsible for the cardioprotection mediated by IP and other pretreatments.

## Introduction

The irreversible myocardial damage that occurs after prolonged ischemia–reperfusion is thought to be mediated, at least in part, by opening of the mitochondrial permeability transition pore (mPTP). Direct pharmacological inhibition of mPTP opening by cyclosporin A (CsA) or sanglifehrin A is cardioprotective, whereas other cardioprotective strategies such as ischemic preconditioning (IP) or temperature preconditioning prevent mPTP opening indirectly.^1^ The mechanism by which IP reduces mPTP opening remains controversial,^1–3^ but our data suggest that a reduction in oxidative stress, a key stimulus of mPTP opening, is of major importance.^4–5^ The cause of oxidative stress is thought to involve increased reactive oxygen species (ROS) production by mitochondria and/or decreased ROS scavenging.^6–7^ Recent work from our laboratory has demonstrated that increased ROS production by mitochondria can be explained, at least in part, by cytochrome c loss from the intermembrane space (IMS) during ischemia.^8^ Oxidized cytochrome c is an excellent scavenger of superoxide, and its loss will lead to greater release of superoxide into the IMS and its subsequent dismutation to hydrogen peroxide. Cytochrome c loss will also restrict electron flow from complex 3 to cytochrome c oxidase, causing the upstream complex 1 to become more reduced and thus to increase matrix superoxide production.^8^

The mechanism of cytochrome c loss from the mitochondria during ischemia remains unclear. Borutaite et al^9^ proposed that mPTP opening might be responsible, but our data do not support this.^8^ Thus, the deoxyglucose entrapment technique did not detect mPTP opening in situ at the end of ischemia, and this is consistent with the inhibition of mPTP opening at the low pH values occurring in ischemia.^10^ Furthermore, unlike Borutaite et al, we were unable to prevent the release of cytochrome c when mPTP opening was inhibited with CsA. However, after ischemia, we did observe a decrease in the content of the antiapoptotic protein, Bcl‐x_L_, in the outer mitochondrial membrane (OMM) of heart mitochondria,^8^ and, in confirmation of work by others,^11^ we also detected a decrease in mitochondria‐bound hexokinase 2 (mtHK2).^8^ It was proposed that the loss of Bcl‐x_L_ might prevent cytochrome c release mediated by endogenous Bax present in the OMM and so unmask a latent cytochrome c permeability pathway.^8^ This would be consistent with recent data from others that show Bax/Bak knockout mice to be resistant to reperfusion injury.^12^ The decrease in mtHK2 may also play a significant role, because several studies have shown that mitochondria with bound HK2 are more resistant to mPTP opening.^13–15^ Furthermore, others have demonstrated that IP prevents the majority of mtHK2 loss during ischemia.^11^ This would be consistent with a role for mtHK2 in preventing cytochrome c loss, thus reducing oxidative stress and mPTP opening.

In this article, we investigate the relative importance of mtHK2 dissociation and Bcl‐x_L_ degradation in mediating cytochrome c loss and thus reperfusion injury. We confirm that IP prevents the majority of mtHK2 loss during ischemia but does not prevent a decrease in Bcl‐x_L_. Furthermore, using a variety of interventions that increase or decrease reperfusion injury, we reveal a strong correlation between the loss of mtHK2 at the end of 30 minutes of ischemia and the infarct size after 120 minutes of reperfusion. We provide data to suggest that this may be explained by the ability of bound HK2 to prevent the breakage of mitochondrial contact sites between the inner mitochondrial membrane (IMM) and the OMM during ischemia.

## Methods

### Antibodies and Chemicals

The following antibodies were used in this study: hexokinase 2 (HK, rabbit monoclonal, Cell Signaling), adenine nucleotide translocase (ANT—raised in‐house against the whole ANT), activated Bax (anti‐Bax YTH‐6A7 monoclonal, Trevigen), and Bcl‐x_L_ (Bcl‐x_L_ [54H6] rabbit monoclonal 2764, Cell Signaling). All chemicals used in this study were purchased from Sigma unless otherwise stated.

### Heart Perfusion

**Figure 1.**
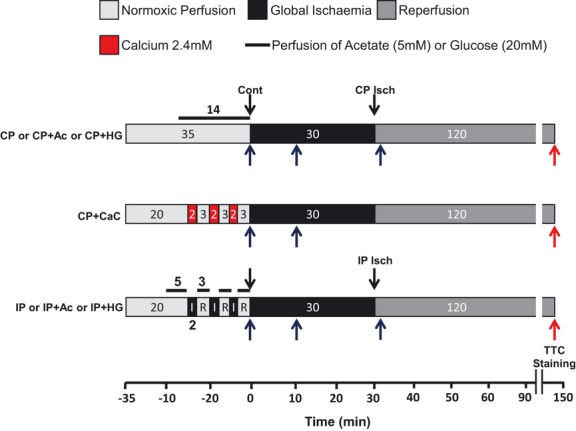
Scheme summarizing the perfusion protocol used. Hearts were Langendorff‐perfused and at the required time removed from the perfusion cannula for the preparation of mitochondria or permeabilized fibers (black arrows) and for staining to assess infarct size (red arrows), or freeze‐clamped (blue arrows). Ac indicates sodium acetate 5 mmol/L; HG, high glucose 20 mmol/L; CaC, calcium challenge 2.4 mmol/L; IP, ischemic preconditioning; CP, control; Cont, heart perfused for 35 minutes; CP Isch, ischemic heart; IP Isch, preconditioned ischemic heart; TTC, triphenyltetrazolium chloride.

All procedures conformed to the UK Animals (Scientific Procedures) Act 1986 and the *Guide for the Care and Use of Laboratory Animals* published by the National Institutes of Health (NIH Publication No. 85‐23. revised 1996). Male Wistar rats (225 to 300 g) were killed via stunning and cervical dislocation, and hearts (≈0.75 g) were rapidly removed into ice‐cold Krebs–Henseleit buffer containing (in mmol/L) NaCl 118, NaHCO_3_ 25, KCl 4.8, KH_2_PO_4_ 1.2, MgSO_4_ 1.2, glucose 11, and CaCl_2_ 1.2, gassed with 95% O_2_/5% CO_2_ at 37°C (pH 7.4). Langendorff heart perfusions were performed as described previously.^16^ Hearts were perfused in a constant flow mode (12 mL/min) according to the protocol schematically described in Figure 1. Global normothermic ischemia (index ischemia) was induced by halting perfusion for 30 minutes and immersing the heart in perfusion buffer at 37°C. At the end of the preischemic, ischemic, or reperfusion period, the hearts were either removed from the perfusion cannula for the preparation of mitochondria and permeabilized fibers, stained to assess infarct size, or freeze‐clamped using liquid nitrogen–cooled tongs. In the last case, hearts were ground under liquid nitrogen and stored at −80°C for later analysis (see later).

### Assessment of Infarct Size

At the end of the reperfusion period, hearts were stained with TTC as described previously^17^ with slight modification. Briefly, hearts were perfused for 2 minutes at 10 mL/min with a 1% (w/v) TTC solution. Hearts were then detached from the cannula and incubated for an additional 5 minutes at 37°C before being sliced perpendicular to the longitudinal axis into 6 slices. The slices were then fixed in 4% (w/v) formalin solution overnight at 4°C and weighed. Both sides of each slice were photographed. The surfaces of the necrotic and area at risk of each side for each slice were determined by planimetry (AlphaEase v5.5), and because global ischemia was used, infarct size was expressed as a percentage of the total cross‐sectional area of the heart.

### Isolation of Mitochondria

Two different protocols were used for mitochondrial preparation involving either Polytron tissue homogenization or protease treatment followed by Dounce Potter homogenization.^8^ The latter gave more mitochondria with less loss of cytochrome c and was used for functional assays. However, this technique was not suitable for determining proteins bound to the OMM because of their degradation by the protease treatment. In both cases, all steps were performed at 4°C.

#### Protease method

Each heart was rapidly chopped into fine pieces with scissors before incubation at 4°C for 7 minutes with stirring in 25 mL of isolation buffer (ISA: sucrose 300 mmol/L, EGTA 2 mmol/L, and Tris‐HCl 10 mmol/L, pH 7.1 at 4°C) containing 0.1 mg/mL bacterial proteinase type XXIV (Sigma). The resulting tissue suspension was poured into a 50‐mL glass Potter homogenizer and homogenized for 3 minutes using a motorized Teflon pestle. The homogenate was centrifuged at 7500*g* for 7 minutes, and the resulting pellet was rinsed twice with 5 mL of ISA, resuspended in 20 mL of ISA, and subjected to additional homogenization as described earlier. The homogenate was then centrifuged at 700*g* for 10 minutes, and the resultant supernatant was centrifuged at 7000*g* for 10 minutes to yield a crude mitochondrial pellet that was resuspended in ISA containing 25% (w/v) Percoll (pH 7.1 to 7.2 at 4°C) and centrifuged at 17 000*g* for 10 minutes. The resulting pellet was resuspended in ISA and centrifuged again at 7000*g* for 10 minutes. The final purified mitochondrial pellet was resuspended in ISA, and the protein concentration was determined by the Biuret method using BSA as a standard. Mitochondria were kept on ice at a final concentration of 50 mg/mL for no longer than 4 hours.

#### Polytron method

All steps were performed essentially as described previously^18^ with slight modification. Each heart was homogenized at 4°C in 6 mL of ISA using a Polytron tissue disruptor (Kinematica) at 10 000 rpm with 2 bursts of 5 seconds and 1 burst of 10 seconds. The homogenate was diluted with 3 volumes of ISAPP (ISA supplemented with inhibitors of proteases [Roche complete] and phosphatases [Sigma cocktail 1]) and further homogenized for 2 minutes in a 50‐mL glass Potter homogenizer using a motorized Teflon pestle. The homogenate was centrifuged at 7500*g* for 7 minutes and the resulting pellet rinsed twice with 5 mL of ISA, resuspended in 20 mL of ISA, and subjected to additional homogenization for 3 minutes as described earlier. The homogenate was then centrifuged at 700*g* for 10 minutes, and the resultant supernatant was centrifuged at 7000*g* for 10 minutes to yield a crude mitochondrial pellet that was resuspended in ISAPP containing 25% (w/v) Percoll (pH 7.1 to 7.2 at 4°C) and centrifuged at 17 000*g* for 10 minutes. The resulting pellet was resuspended in ISAPP and centrifuged again at 7000*g* for 10 minutes. The final purified mitochondrial pellet was resuspended in ISAPP, and the protein concentration was determined by the Biuret method using BSA as a standard. Mitochondria were kept on ice at a final concentration of 50 mg/mL or stored at −80°C for later analysis.

### Preparation of Permeabilized Cardiac Fibers

Preparation of permeabilized cardiac left ventricular fibers was performed using well‐established protocols.^19–21^ Small pieces of cardiac muscle were taken from the left ventricle to prepare permeabilized fibers at different points of the perfusion protocols as shown in Figure 1 (black arrows). All procedures were carried out at 4°C. The samples were rapidly dissected into bundles of fibers and incubated with stirring in 3 mL of solution A (see later) containing saponin (50 μg/mL) before washing twice for 10 and 20 minutes in solution B (see later).

#### Solution A

Solution A contains (in mmol/L) CaK_2_EGTA 2.77, K_2_EGTA 7.23 (pCa=7), MgCl_2_ 6.56, DTT 0.5, MES 50, imidazole 20, taurine 20, Na_2_ATP 5.3, and creatine phosphate 15. pH was adjusted to 7.3 (4°C) with KOH 10 mol/L.

#### Solution B

Solution B contains (in mmol/L) CaK_2_EGTA 2.77, K_2_EGTA 7.23 (pCa=7), MgCl_2_ 1.38, DTT 0.5, MES 100, imidazole 20, taurine 20, and KH_2_PO_4_ 3. pH was adjusted to 7.1 (4°C) with KOH 10 mol/L and BSA 2 mg/mL added.

### Hexokinase and Citrate Synthase Specific Activity

Aliquots (0.75 mg of protein) of frozen mitochondria prepared through the Polytron method (see earlier) were solubilized via brief sonication at 4°C in a buffer containing KH_2_PO_4_ 33 mmol/L and DTT 50 μmol/L (pH 7.2 at room temperature), and the concentration was adjusted to 2 mg/mL. For assay of mtHK isoforms 1 and 2, samples (20, 30, or 40 μL) were added to 1 mL of assay buffer (pH 7.4 at room temperature) containing Tris‐HCl 100 mmol/L, NADP^+^ 0.4 mmol/L, MgCl_2_ 10 mmol/L, ATP 5 mmol/L, Triton X‐100 0.3% (v/v), and glucose‐6‐phosphate dehydrogenase (G‐6‐P) 0.5 U/mL and incubated for 2 minutes at 37°C before the addition of glucose (1 mmol/L final) to start the reaction. Hexokinase activity was calculated from the rate of NADPH production corrected for glucose‐independent rates of NADPH formation determined in parallel assays lacking glucose. For assay of citrate synthase, mitochondrial samples (20, 30, or 40 μL of 0.2 mg protein/mL) were added to 1 mL of assay buffer (pH 7.4 at room temperature) containing Tris‐HCl 50 mmol/L, Triton X‐100 0.3% (v/v), and 5,5′‐dithiobis‐2‐nitrobenzoic acid (DTNB) 150 μmol/L and incubated for 2 minutes in the presence of acetyl‐coenzyme A 0.3 mmol/L, before the addition of oxaloacetic acid (500 μmol/L final) to start the reaction. One unit of citrate synthase was defined as equal to the utilization of 1 μmol of DTNB per minute.

Hexokinase and citrate synthase specific activities were also determined in permeabilized cardiac fibers. Frozen fibers were solubilized in a 1‐mL Potter homogenizer at 4°C in a buffer containing EDTA 1 mmol/L, Triton X‐100 0.5% (w/v) , and KH_2_PO_4_ 50 mmol/L, pH 7.2 at room temperature with KOH. The procedure used was the same as described earlier except that for the citrate synthase assay, the samples were diluted 20 times.

### Measurement of Mitochondrial Bcl‐xl and Hexokinase by Western Blotting

Mitochondria prepared by the Polytron method (see earlier) were separated via SDS‐PAGE (12% for Bcl‐xl and ANT, 5% for hexokinase) using 20 μg of protein for each track. Gels were then subjected to Western blotting with the required primary antibody (see earlier), and blots were developed using the required immunoglobulin horseradish peroxidase secondary antibody, with ECL/ECL^+^ detection (Amersham Biosciences UK Limited). Appropriate exposures of the film were used to ensure that band intensities were within the linear range. Quantification of blots was performed using an AlphaInotech ChemiImager 4400 to image the blot and analysis of band intensity with AlphaEase v5.5 software. Each blot contained samples of control and end‐ischemic mitochondria to allow direct comparisons between groups using the same film exposure. To normalize band intensities, parallel blots were performed on the same samples using antibodies against the ANT.

### Preparation of Freeze‐Clamped Heart Powder Deproteinized Sample

Frozen heart powder (150 to 200 mg) was added to 2 mL of ice‐cold perchloric acid (PCA, 0.3 mol/L final) and Polytron‐homogenized on ice for 3 bursts at 10 000 rpm of 5 seconds interspersed by 10 seconds. The resulting homogenate was further homogenized in a 2‐mL glass Potter until complete dissolution using a motorized Teflon pestle. The homogenate was then centrifuged for 10 minutes at 4000*g*. The resulting supernatant was neutralized with KHCO_3_. The solution obtained was then centrifuged for 10 minutes at 4000*g*, and the supernatant was stored at −80°C for later analysis.

### G‐6‐P, l‐Lactate, Phosphocreatine, ATP, and Glycogen Assay in Deproteinized Heart Powder Samples

To determine the G‐6‐P content in deproteinized samples (see earlier), 200 μL was incubated for 1 minute at 37°C in 1 mL final of assay buffer (Tris‐HCl 100 mmol/L, NADP^+^ 0.4 mmol/L, pH 7.4 at room temperature). The reaction was started by the addition of 0.5 U/mL of G‐6‐P dehydrogenase and stopped when the A_340_ reached a plateau. A standard curve performed in the same buffer in the presence of known G‐6‐P was used to express the G‐6‐P content of the deproteinized samples given in μmol per gram wet weight. To determine l‐lactate content in deproteinized samples, 200 μL was incubated for 2 to 3 hours at 37°C in 1 mL final of buffer containing glycylglycine 0.27 mol/L, hydrazine hydrate 0.7 mol/L, NAD^+^ 0.4 mmol/L, and l‐lactate dehydrogenase 1 U/mL, pH 8 at room temperature. A_340_ was read every 30 minutes, and the reaction was stopped when it reached a stable value. Parallel experiments were performed in the absence of lactate dehydrogenase to evaluate lactate‐independent changes in A_340_ over time. A standard curve was performed under identical conditions in the presence of known concentrations of l‐lactate. l‐Lactate content in the deproteinized samples was expressed as μmol per gram wet weight. Glycogen content in deproteinized samples was measured using a method adapted from Passonneau et al.^22^ Aliquots (100 mg) of frozen heart powder were weighed and added to 1 mL of perchloric acid (0.3 mol/L), Polytron‐homogenized for 10 seconds, and transferred to a 2‐mL Dounce Potter for further homogenization (20 passes). Aliquots (50 μL) of the homogenate were added to 500 μL of a buffer containing Na^+^‐acetate 50 mmol/L and BSA 0.02% (w/v), pH 5.5. Amyloglucosidase (AG) was then added at a final concentration of 50 μg/mL, and the samples were incubated 2 hours with agitation at room temperature. Parallel samples were run without AG to determine basal glucose concentration. Samples were then centrifuged for 10 minutes at 16 000*g*, and the supernatant was stored at −80°C. To evaluate the glucose content of the sample, 400 μL was incubated in assay buffer containing Tris‐HCl 100 mmol/L, DTT 0.6 mmol/L, MgCl_2_ 10 mmol/L, NADP^+^ 0.4 mmol/L, ATP 5 mmol/L, and BSA 0.04% (w/v), pH 8 at room temperature. The reaction was started by the addition of hexokinase 0.25 U/mL and G‐6‐P dehydrogenase 0.5 U/mL. The reaction was continued for 20 minutes with one reading of A_340_ taken every 10 minutes. Parallel experiments were performed without samples in the presence of known concentration of glucose, and glycogen content was expressed as μmol of glucose per gram wet weight.

To determine phosphocreatine (PCr) content in deproteinized samples (see earler), 100 μL was incubated at 37°C in assay buffer (Tris‐HCl 100 mmol/L, MgCl_2_ 10 mmol/L, glucose 2 mmol/L, ADP 1 mmol/L, hexokinase 1.2 U/mL, G‐6‐P dehydrogenase 2 U/mL, and NADP^+^ 1 mmol/L, pH 7.4 at room temperature) until the A_340_ reached a plateau. The reaction was then started by the addition of 40 μg of creatine kinase (from rabbit muscle, Roche) and stopped when the plateau was reached. ATP content was determined in the same buffer lacking hexokinase. After the addition of samples (50 μL) and stabilization of A_340_, the reaction was started by the addition of 1.2 units of hexokinase and stopped when the plateau was reached. Internal calibration was performed for each sample by successive addition of 2 known concentration of PCr or ATP. PCr and ATP content was expressed as μmoles/g wet weight.

### Measurements of Respiration

Oxygen consumption by permeabilized skinned fibers was measured at 37°C using an Oroboros Oxygraph (Graz, Austria) at 37°C in a buffer containing KCl 125 mmol/L, MES 100 mmol/L, KH_2_PO_4_ 3 mmol/L, MgCl_2_ 3 mmol/L, and taurine 20 mmol/L, pH 7.3 at room temperature with KOH. Unless stated otherwise, the respiratory substrate was a mixture of l‐glutamate 10 mmol/L, l‐malate 4 mmol/L, and succinate 10 mmol/L (GMS) to ensure electron supply at both complex I and complex II of the respiratory chain as occurs in vivo.^8^ Rates of respiration were determined after the addition of ADP 2 mmol/L (state 3) or carboxyatractyloside (CAT) 5 μmol/L as a measure of respiration independent of ATP export. The permeability of the OMM to cytochrome c was assessed by the addition of exogenous cytochrome c (12.5 μmol/L) after ADP or CAT. At the end of the run, fibers were dried and oxygen consumption was expressed in nmol O_2_/min per milligram of dry weight.

Oxygen consumption of isolated mitochondria was measured at 37°C in a buffer containing KCl 125 mmol/L, MOPS 20 mmol/L, Tris 10 mmol/L, EGTA 10 μmol/L, KH_2_PO_4_ 2.5 mmol/L, and MgCl_2_ 2.5 mmol/L, pH 7.3 at room temperature with KOH. Rates of respiration were determined before and after the addition of ADP 1.5 mmol/L (state 2 and state 3, respectively) in the presence of GMS. The permeability of the mitochondrial outer membrane to cytochrome c was assessed by the addition of exogenous cytochrome c (10 μmol/L) after ADP.

### H_2_O_2_ Formation in Isolated Mitochondria and Permeabilized Fibers

The rate of H_2_O_2_ production in isolated mitochondria was determined with the fluorescent hydrogen peroxide indicator Amplex Red (30 μmol/L) in the presence of peroxidase (10 μg/mL) in the KCl‐based respiration buffer (see earlier) at 37°C as described previously.^8^ H_2_O_2_ accumulation in permeabilized fibers was monitored at 37°C in a KCl‐based buffer (see earlier) supplemented with Amplex Red (30 μmol/L) and in the presence of peroxidase (10 μg/mL). Fibers were incubated for 10 minutes with ADP 2 mmol/L in the absence or presence of cytochrome c 12.5 μmol/L as indicated in the legends to the relevant figures. At the end of each run, 1 mL of the respiration buffer was frozen in liquid N_2_ and the resultant formation of resofurin was studied in a multiwell fluorescence plate reader (Flexstation, Molecular Devices) using excitation and emission wavelengths at 540 and 585 nm, respectively. Parallel blanks were performed in the absence of fibers over the same length of time with the same additions to evaluate the background formation of resofurin. Calibration curves were performed in the absence and presence of cytochrome c as described previously.^8^

### In Vitro Dissociation of Hexokinase 2 in Permeabilized Cardiac Fibers

After incubation in solution A (see earlier), fibers were washed twice for 10 and 20 minutes in solution B containing G‐6‐P 10 mmol/L. Solution B and G‐6‐P stock solution were brought to the required pH value at 4°C by the addition of HCl. Fibers were then stored in solution B at pH 7.1 (4°C) in the absence of G‐6‐P. In a separate group of experiments, fibers were washed in solution B at pH 6.3 (4°C) in presence of different G‐6‐P concentrations from 1 to 10 mmol/L as indicated in the relevant figure legend.

### G‐6‐P, PCr, and ATP Accumulation in Permeabilized Fibers

To determine G‐6‐P accumulation, fibers (0.5 to 1 mg dry weight) were incubated at 37°C for 5 minutes in 2 mL final of solution B supplemented with GMS, ADP 1 mmol/L, and glucose 5 mmol/L. To study PCr and ATP accumulation, fibers were incubated for 5 minutes at 37°C in 2 mL final of solution B supplemented with GMS, ADP 1 mmol/L, and creatine 10 mmol/L in the presence or absence of CAT 5 μmol/L. In a separate group of experiments, PCr and ATP accumulation was measured in the additional presence of glucose 5 mmol/L as indicated in the relevant figure legend. At the end of each run, the fibers were removed and dried, and the resultant solution was incubated for 10 minutes at 4°C in PCA (0.3 mol/L final). The samples were then stored at −80°C for later analysis of their content of G‐6‐P, PCr, and ATP as described later.

### G‐6‐P, PCr, and ATP Determination in Samples From Permeabilized Fibers

The samples obtained from the incubation of permeabilized fibers (see earlier) were thawed and neutralized with KHCO_3_ before assay. To assay G‐6‐P content, 500 μL of sample was incubated at 37°C in a buffer (1 mL final) containing Tris‐HCl 100 mmol/L (pH 7.4 at room temperature), MgCl_2_ 10 mmol/L, and NADP^+^ 1 mmol/L. After 1 minute of incubation, G‐6‐P dehydrogenase was added (2 U/mL final) and the reaction was stopped when the A_340_ was stable. A standard curve performed with known G‐6‐P concentration under the same conditions was used to express the G‐6‐P content as nmoles/min per milligram of dry weight. To determine PCr content, 200 μL was incubated for 4 minutes at 37°C in a buffer (1 mL final) containing Tris‐HCl 100 mmol/L, MgCl_2_ 10 mmol/L, glucose 2 mmol/L, ADP 1 mmol/L, hexokinase 1.2 U/mL, G‐6‐P dehydrogenase 2 U/mL, and NADP^+^ 1 mmol/L, pH 7.4 at room temperature. The reaction was then started by the addition of 40 μg of creatine kinase (from rabbit muscle, Roche) and stopped when the A_340_ was stable. ATP content was determined in the same buffer lacking hexokinase. After the addition of 100 μL samples and stabilization of the A_340_, the reaction was started by the addition of 1.2 U/mL hexokinase and stopped when the plateau was reached. Internal calibration was performed for each sample by the successive addition of 2 known concentrations of PCr or ATP. PCr and ATP contents were expressed as nmoles/min per milligram of dry weight.

### Statistical Analysis

Data are presented as mean±SEM. Comparison among several groups were made with a 1‐way ANOVA followed by a Student–Newmann–Keuls post‐hoc test (Kaleidagraph, 4.03 software). One‐way ANOVA *P* values are indicated in the figure legends. An Omnibus Normality test (NCSS2000, software) of the ANOVA residuals and a modified Levene equal‐variance test were performed to confirm ANOVA assumptions (normality and homoscedasticity).

When assumptions tests were met, the comparison among 2 independent groups or comparison of paired data among the same group was made with use of the corresponding Student *t* test. In the latter case, Student *t* test was used to perform only one comparison between 2 groups. Consequently, no further correction was applied.

When ANOVA assumptions tests were rejected, comparison among several groups were made by Kruskal–Wallis rank sum test (*P* values indicated in the figure legends) and followed by a Mann–Whitney rank sum test with Bonferroni correction for comparison between multiple groups. For comparison of independent groups (1‐way ANOVA or Mann–Whitney), *P* values are indicated on the figure. For comparison of dependent groups or comparison of paired groups, *P* values are indicated in the figure legends. Differences were considered significant for a *P* value <0.05 regardless of the statistical analysis performed.

## Results

### IP Prevents Permeabilization of the OMM During Ischemia and the Resulting H_2_O_2_ Production Caused by Cytochrome c Loss

**Figure 2.**
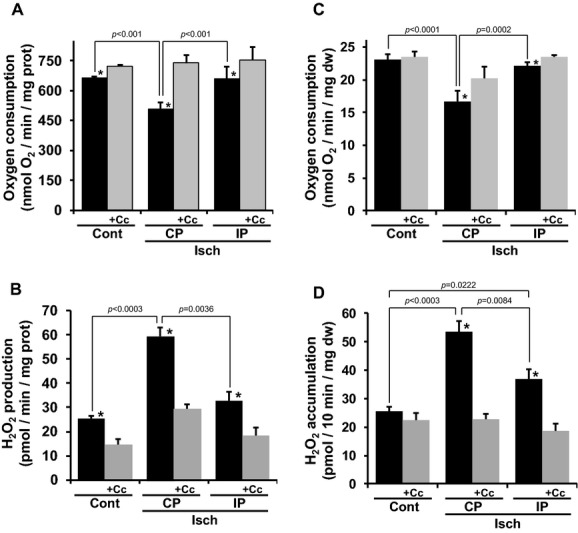
IP prevents OMM permeabilization and decreases mitochondrial H_2_O_2_ production. Three groups of heart were studied as described in Figure 1. Isolated mitochondria or permeabilized fibers were prepared and incubated in the presence of ADP. (A and C) Oxygen consumption and (B and D) H_2_O_2_ production of isolated mitochondria and permeabilized fibers respectively in the absence (black bar) or presence (gray bar, +C*c*) of exogenous cytochrome c. Data are presented as means±SEM. (A) Cont (n=5), CP Isch (n=18), IP Isch (n=6), ANOVA *P*=0.002 for groups without C*c*, *P<0.05 vs paired control +C*c*. (B) Cont (n=10), CP Isch (n=14), IP Isch (n=5), Kruskal–Wallis *P*<0.0001 for groups without C*c*, *P<0.05 vs paired control +C*c*. (C) Cont (n=12), CP Isch (n=8), IP Isch (n=9), ANOVA *P*<0.0001 for groups without C*c*, *P<0.05 vs paired control +C*c*. (D) Cont (n=10), CP Isch (n=9), IP Isch (n=6), Kruskal–Wallis *P*<0.0001, *P<0.003 vs paired control +C*c*. Cont indicates normoxic mitochondria or fibers; CP Isch, ischemic mitochondria or fibers; IP Isch, preconditioned ischemic mitochondria or fibers; OMM, outer mitochondrial membrane.

Control and IP hearts were perfused according to the protocol described in Figure 1, and mitochondria or permeabilized fibers were prepared before or after 30 minutes of ischemia. As reported previously,^8^ after ischemia, mitochondria or fibers isolated from control hearts showed a decreased rate of respiration compared with the preischemic group, but these changes could be reversed by the addition of exogenous cytochrome c (Figure 2A and 2B). By contrast, mitochondria from the IP ischemic group showed little reduction in the rate of respiration or subsequent effect of cytochrome c addition. Similarly, H_2_O_2_ production by isolated mitochondria or permeabilized fibers was increased after ischemia, but this was prevented in the mitochondria from the IP ischemic group (Figure 2C and 2D). The addition of exogenous cytochrome c largely reversed the increased H_2_O_2_ production in the ischemic mitochondria or fibers. It should be noted that even in mitochondria from normoxic hearts, there is a small effect of exogenous cytochrome c on respiration and H_2_O_2_ production that is not seen in permeabilized fibers. This most likely reflects a slight damage to the OMM during mitochondrial preparation.^8^ Taken together, these data imply that IP reduces OMM permeabilization and consequent cytochrome c loss during ischemia, leading to less inhibition of respiration and H_2_O_2_ production.

### OMM Permeabilization Cannot be Explained Simply by Bcl‐x_L_ Loss or Bax Activation

**Figure 3.**
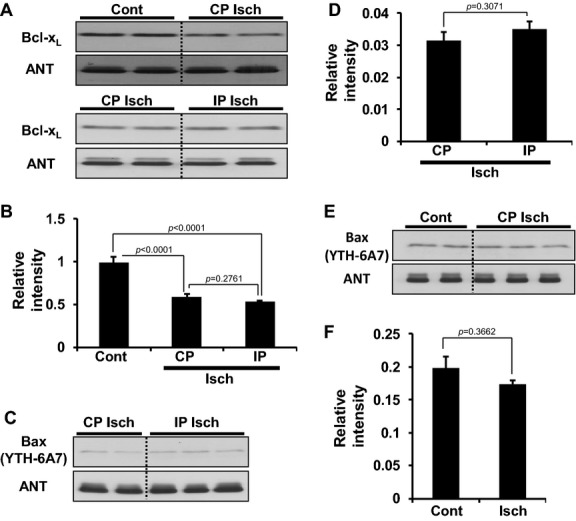
Bcl‐X_L_ cleavage and Bax activation during ischemia. Typical Western blots for Bcl‐x_L_ (A) and activated Bax YTH‐6A7 (C and E) of isolated mitochondria from each group of hearts (see Figure 1). (B, D, and F) Ratio of Bcl‐x_L_ or activated Bax (YTH‐6A7) to ANT derived from scanning the blots. Data are presented as means±SEM, n=5 for each group. (B) ANOVA *P*<0.0001. Cont indicates normoxic mitochondria; CP Isch, ischemic mitochondria; IP Isch, preconditioned ischemic mitochondria; ANT, adenine nucleotide translocase.

**Figure 4.**
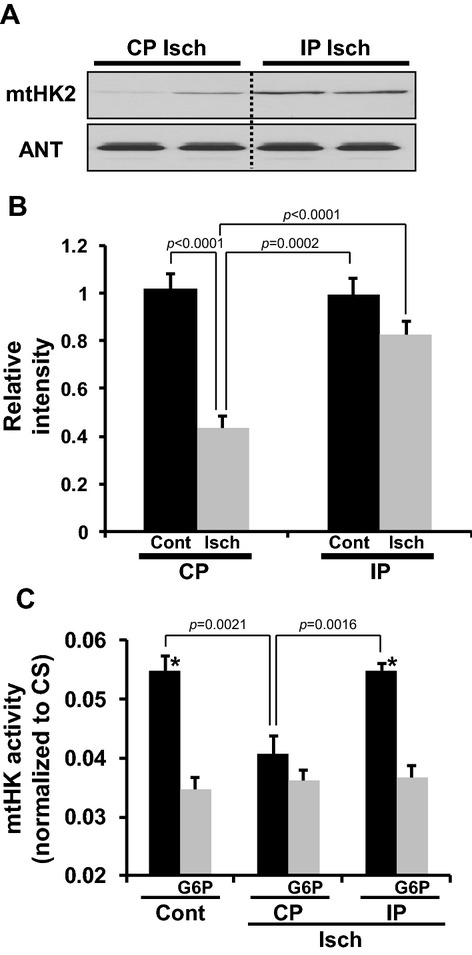
IP prevents mtHK2 dissociation during ischemia. (A) Typical Western blots for mtHK2 and ANT of mitochondria isolated from 4 groups of hearts (see Figure 1). (B) Ratio of mtHK2 to ANT derived from scanning the blots. (C) mtHK activity of permeabilized fibers washed at pH 6.3 (4°C) from 3 different groups of hearts in the absence (black bar) or presence of 10 mmol/L G‐6‐P (gray bar, G6P). Data are presented as means±SEM. (A) CP Cont (n=5), CP Isch (n=12), IP Cont (n=5), and IP Isch (n=12). (C) Cont (n=9), CP Isch (n=6), and IP Isch (n=6). (B) ANOVA *P*<0.0001. (C) ANOVA *P*=0.0013 for groups without G‐6‐P, *P<0.05 vs corresponding control +G‐6‐P. mtHK2 indicates mitochondria‐bound hexokinase 2; ANT, adenine nucleotide translocase; Cont, normoxic mitochondria; CP Isch, ischemic mitochondria; IP Isch, preconditioned ischemic mitochondria; G‐6‐P, glucose‐6‐phosphate.

Translocation and/or activation of Bcl2 family members is known to play a key role in OMM permeabilization, and it has recently been shown that the deletion of Bax and Bak dramatically reduces necrotic injury during myocardial infarction in vivo.^12^ We previously showed that neither Bax nor Bak levels increased in mitochondria after ischemia, but there was a decrease in the antiapoptotic protein Bcl‐x_L_ that might account for OMM permeabilization.^8^ The data of Figure 3A and 3B confirm this loss but reveal that it was not prevented by IP. Nor did IP have any effect on the level of Bax detected with an antibody directed against a region of Bax that is only revealed after its activation,^23^ as shown in Figure 3C through 3F. These results indicated that neither Bcl‐x_L_ content nor Bax activation was directly responsible for the end‐ischemic OMM permeabilization. However, as reported by Gurel et al,^11^ IP did prevent the loss of mtHK2 after ischemia, measured with both Western blotting (Figure 4A and 4B) and enzymic activity (Figure 4C). This suggested that mtHK2 might regulate OMM permeabilization, and we decided to investigate this further.

### mtHK2 Content at the End of Ischemia Strongly Correlates With the Infarct Size

**Figure 5.**
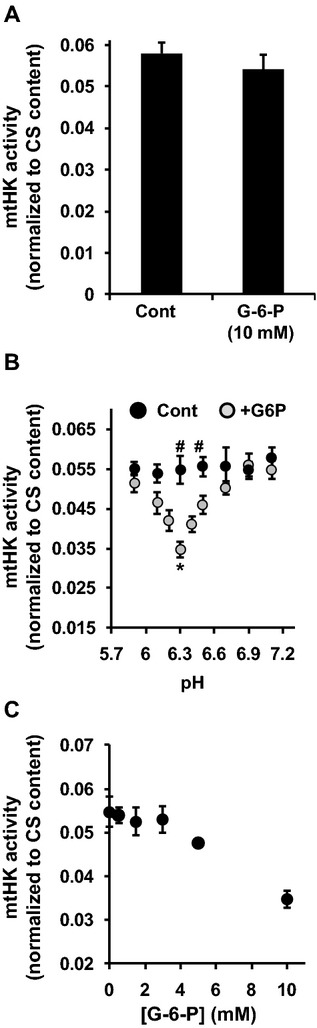
Effect of pH and G‐6‐P on mtHK dissociation in permeabilized fibers. (A) Total activity of bound mitochondrial hexokinase isoforms 1 and 2 (mtHK) normalized to citrate synthase content (CS) assessed in permeabilized fibers. Fibers were prepared from a normoxic perfused heart and washed twice in the absence (Cont) or presence of glucose‐6‐phosphate (G‐6‐P, or G6P) 10 mmol/L at pH 7.4 (4°C). (B) mtHK activity normalized to CS measured in permeabilized fibers. Fibers were permeabilized and then washed at 4°C in the presence or absence of G‐6‐P 10 mmol/L in solution B at the pH indicated. (C) Effect of increasing [G‐6‐P] on the ratio mtHK to CS monitored on permeabilized fibers washed at pH 6.3 (4°C). After permeabilization fibers were washed twice in solution B at 4°C and pH 6.3 in the presence of increasing concentration of [G‐6‐P]. (A) n=8 in each group. (B) Group Cont: pH 5.9 (n=6), pH 6.1 (n=6), pH 6.3 (n=10), pH 6.5 (n=6), pH 6.7 (n=6), pH 6.9 (n=6), and pH 7.1 (n=8). Group +G‐6‐P: pH 5.9 (n=10), pH 6.1 (n=6), pH 6.2 (n=8), pH 6.3 (n=11), pH 6.4 (8), pH 6.5 (n=9), pH 6.7 (n=10), pH 6.9 (n=6), and pH 7.1 (n=8). ANOVA *P*<0.0001 for groups with G‐6‐P, *P<0.05 vs pH 6.2 +G‐6‐P and pH 6.1 +G‐6‐P, #*P*<0.02 vs corresponding control pH with G‐6‐P. (C) n=3 in each group except for the group [G‐6‐P]=10 mmol/L (n=11).

**Figure 6.**
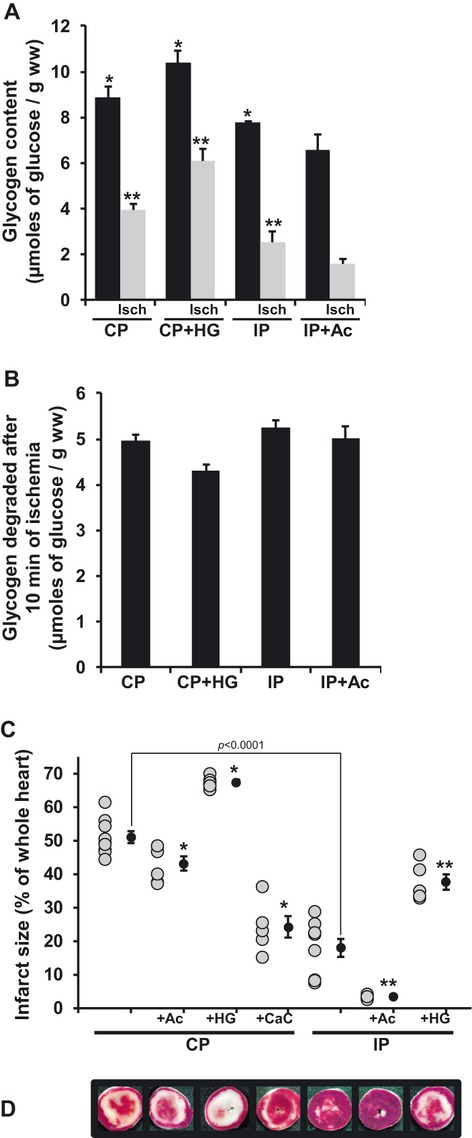
Effect of ischemia on glycogen content, infarct size, and ischemic contracture. (A) Hearts were perfused according to the protocols described in Figure 1 and freeze‐clamped either before ischemia or after 10 minutes of global ischemia (Isch). Their glycogen content was assayed as described in Materials and Methods. (B) Quantity of glycogen broken down after 10 minutes of ischemia derived from the data of (A). (C) Infarct size for the different groups of hearts perfused as described in Figure 1. Gray dots represent individual experiment and black dots represent means±SEM. (D) Typical hearts slices (cut 3) from each group. Data are presented as means±SEM. (A) n=4 in each group, ANOVA *P*=0.0051 among preischemic groups, *P<0.05 vs IP+Ac group, ANOVA *P*<0.0001 among ischemic group, **P<0.05 vs IP+Ac Isch group. (C) CP (n=9), CP+Ac (n=6), CP+HG (n=6), CP+CaC (n=5), IP (n=9), IP+Ac (n=6) and IP+HG (n=5). ANOVA *P*<0.001 among all groups, *P<0.05 vs CP; **P<0.05 vs IP. CP indicates control; Ac, sodium acetate; HG, high glucose; CaC, calcium challenge; IP, ischemic preconditioning.

**Figure 7.**
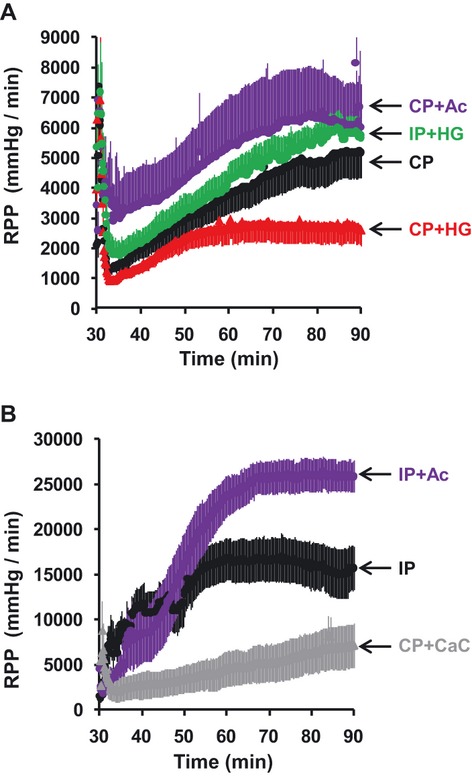
Rate‐pressure product (RPP) recovery during the first 60 minutes of reperfusion. (A) RPP recovery (mm Hg/min) of hearts for which infarct size was above 35% of the whole heart (see Figure 6C). (B) RPP recovery (mm Hg/min) of hearts for which infarct size was <35% of the whole heart (see Figure 6C). Data are presented as means±SEM. (A and B) CP (n=9), CP+Ac (n=6), CP+HG (n=6), CP+CaC (n=5), IP (n=9), IP+Ac (n=6) and IP+HG (n=5). (A and B) SEM is reflected by the “thickness” of the plotted lines. See Figure 1 for details of the perfusion protocols used in each group. See Tables 1 through 3 for a detailed description of the hemodynamic function during the preischemic, reperfusion, and ischemic phases, respectively. CP indicates control; Ac, sodium acetate; HG, high glucose; CaC, calcium challenge; IP, ischemic preconditioning.

**Figure 8.**
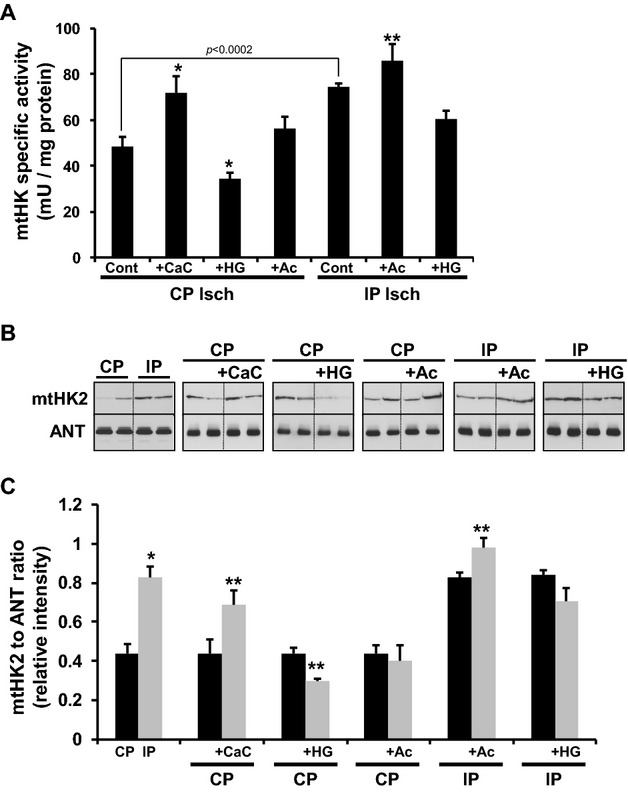
Effect of ischemia following different preischemic interventions on mtHK activity and mtHK2 content. Hearts were perfused according to the protocols described in Figure 1 and submitted to 30 minutes of global ischemia. Mitochondria were isolated (Polytron method) and purified at the end of the ischemic period as described in Materials and Methods. (A) The total hexokinase activity (isoforms 1 and 2, mtHK) of isolated purified mitochondria for each group. (B) Typical Western blots performed on the corresponding samples (A) for the mitochondrial isoform 2 (mtHK2) and ANT. (C) The ratio of mtHK2 to ANT derived from scanning the blots. Data are presented as means±SEM n=5 for each group. (A) ANOVA *P*<0.0001 among all groups, *P<0.05 vs CP Isch Cont, **P<0.05 vs IP Isch Cont. (C) * *P*<0.05 vs IP, ** *P*<0.05 vs corresponding control. CP indicates control; Ac, sodium acetate; HG, high glucose; CaC, calcium challenge; IP, ischemic preconditioning; mtHK2, mitochondria‐bound hexokinase 2.

**Figure 9.**
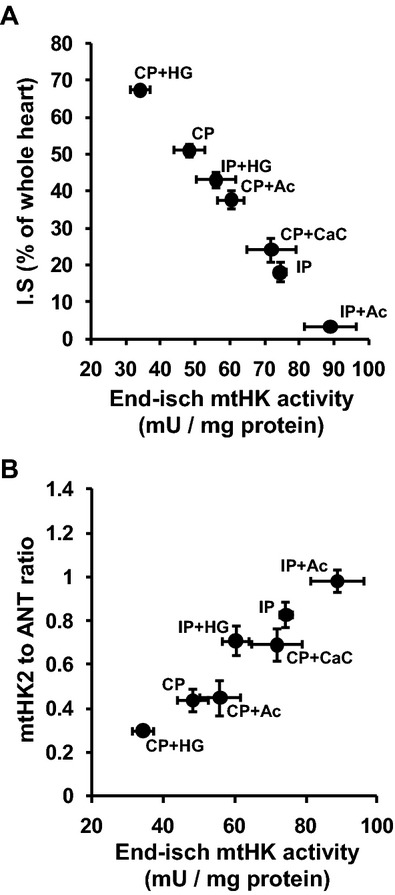
End‐ischemic mtHK2 content correlates with infarct size. Heart were perfused as described in Figure 1. End‐ischemic mtHK activity and mtHK2 content were studied on isolated purified mitochondria as shown in Figure 8. In a separate group of hearts infarct size was assessed as shown in Figure 6C. (A) Infarct size (n=5 to 9 for each group as noted in Figure 6C) was plotted as a function of end‐ischemic mtHK activity (n=5 for each group). (B) End‐ischemic mtHK2 content (n=5 for each group) as a function of mtHK activity. CP indicates control; Ac, sodium acetate; HG, high glucose; CaC, calcium challenge; IP, ischemic preconditioning; mtHK2, mitochondria‐bound hexokinase 2.

Initially, we attempted to use the TAT‐HK2 peptide that others have shown can dissociate mtHK2 from mitochondria,^24^ but this was found to exert effects on the vasculature of the heart independent of dissociation of mtHK2 from the myocyte mitochondria.^25^ Thus, we looked for an alternative means to modulate mtHK2 binding in situ. In mitochondria isolated from HeLa cells, a 30‐minute preincubation period with G‐6‐P 1 mmol/L was reported to significantly dissociate mtHK2.^26^ We investigated whether this was the case with mitochondria in permeabilized fibers but found no dissociation after incubation with G‐6‐P at concentrations up to 10 mmol/L (Figure 5A). However, mtHK dissociation was obtained when fibers were incubated in the presence of G‐6‐P at pH values <7.0, with maximal dissociation being achieved at pH 6.3 (Figure 5B). At pH 6.3, increasing the concentration of G‐6‐P caused greater dissociation of mtHK (Figure 5C), with no dissociation occurring in the absence of G‐6‐P (Figure 5B, Cont). On the basis of these data, we hypothesized that increased G‐6‐P levels and low pH during ischemia might, at least in part, be responsible for mtHK2 dissociation as considered further in the Discussion. Consequently, hearts were perfused under conditions known to interfere with glucose metabolism during ischemia according to the protocols summarized in Figure 1. To favor glycolysis during ischemia, control and preconditioned hearts were perfused with glucose 20 mmol/L before ischemia. To impair glycolysis during ischemia, control and preconditioned hearts were perfused with acetate 5 mmol/L before the index ischemia.^27^ In 2 separate groups of hearts, the rate of glycolysis was modified by using IP to deplete glycogen before ischemia (Figure 6A and 6B) or by increasing heart workload with elevated calcium. Infarct size (Figure 6C and 6D) and hemodynamic parameters (Tables 1 through 3 and Figure 7) were determined for each group. It has been shown that there is a correlation between the time at which ischemic rigor starts (T_0_) and ischemic glycolysis rate.^28^ Thus, we used the value of T_0_ in the different groups of hearts as an index of glucose metabolism during ischemia and confirmed that the smaller the T_0_, the better was the cardioprotection observed (compare Figure 6C to Table 3). These results are consistent with cardioprotection being associated with an early inhibition of glycolysis and hence decreased G‐6‐P concentration and a smaller drop in pH_i_ leading to reduced mtHK2 dissociation. Measurement of end‐ischemic mtHK activity and mtHK2 content in isolated purified mitochondria supported this conclusion (Figure 8). Furthermore, when infarct size was plotted as a function of the end‐ischemic mtHK activity, a strong inverse correlation was obtained (Figure 9A). We chose HK activity for this correlation because it is more quantitative than Western blotting, but we were able to demonstrate a good linear correlation between total mtHK activity and mtHK2 content (Figure 9B). Overall, these data suggest that the end‐ischemic mtHK2 content is somehow linked to the heart viability during the reperfusion period.

**Table 1.** Hemodynamic Data Monitored Before Ischemia

	n	AP, mm Hg		RPP, mm Hg/min		HR, beats/min		EDP, mm Hg		SP, mm Hg	
		Base	Treatment	Base	Treatment	Base	Treatment	Base	Treatment	Base	Treatment
CP	16	107±5		23 120±1031		294±10		4±1		84±5	
CP+Ac	12	98±6	59±3*	26 206±1781	26 540±1705	316±19	299±7	7±1	7±1	91±5	96±6
CP+HG	12	117±4	122±4	25 236±1489	25 484±1401	289±13	281±12	9±1	10±1	99±6	102±5
CP+CaC	11	107±5	71±6*	27 481±1287	41 751±1969*	306±8	295±7	8±1	7±1	98±4	150±7*
IP	23	98±6	49±2*	24 611±944	16 765±587*	303±5	304±8	7±1	7±1	88±3	63±2*
IP+Ac	13	104±5	48±2*	25 528±1354	13 018±635*	301±9	302±5	11±1	10±1	103±5	54±3*
IP+HG	12	107±8	47±2*	25 855±1494	15 778±761*	300±10	294±8	9±1	9±1	96±5	63±3*

Hearts were perfused according to the protocols described in Figure 1. All the data presented in the table correspond to hemodynamic function recorded prior to the index ischemia at the end of the stabilization period (Base) or at the end of each respective treatment applied (Treatment). AP indicates aortic pressure; RPP, rate pressure product; HR, heart rate; EDP, end‐diastolic pressure; SP, systolic pressure; CP, control; Ac, sodium acetate; HG, high glucose; CaC, calcium challenge; IP, ischemic preconditioning.

* *P*<0.0001 Treatment vs corresponding Base.

* *P*<0.0001 Treatment vs corresponding Base.

* *P*<0.0001 Treatment vs corresponding Base.

**Table 2.** Hemodynamic Data Monitored During Reperfusion for the Group of Hearts Used to Study Infarct Size

	n	AP, mm Hg			HR, beats/min			EDP, mm Hg			SP, mm Hg			AAR, cm^3^
		5 min	30 min	60 min	5 min	30 min	60 min	5 min	30 min	60 min	5 min	30 min	60 min	
CP	9	90±9	101±11	114±13	305±110	305±14	319±15	91±6	62±8	52±5	98±7	71±8	69±8	0.54±0.04
CP+Ac	6	79±4	86±4	92±6	295±43	300±8	320±15	111±6	90±5	83±6	122±5	109±6	103±6	0.51±0.02
CP+HG	6	84±5	96±7	104±6	369±34	303±14	314±13	135±6	97±2	91±3	138±5	107±2	100±4	0.48±0.02
CP+CaC	5	90±5	94±6	102±8	298±21	305±22	285±32	110±4	78±3	71±3	118±5	94±7	91±3	0.42±0.02
IP	9	66±5*	74±2*	86±8*	299±52	296±14	292±9	50±7*	36±7*	36±7*	83±9	90±9	87±8	0.53±0.06
IP+Ac	6	66±4*	72±2*	85±4*	270±29	308±7	305±13	68±7*	28±3*	27±2*	87±9	104±4	112±4	0.45±0.02
IP+HG	5	77±3	92±5*	106±6*	287±37	277±3	274±10	112±5*	87±2*	85±2*	119±5	103±4	106±4	0.50±0.02

Hearts were perfused according to the protocols described in Figure 1. All the data presented in the table correspond to hemodynamic function recorded after 5, 30, and 60 min of reperfusion. AP indicates aortic pressure; HR, heart rate; EDP, end‐diastolic pressure; SP, systolic pressure; AAR, area at risk; CP, control; Ac, sodium acetate; HG, high glucose; CaC, calcium challenge; IP, ischemic preconditioning.

AP 5 min: *P<0.05 vs CP; AP 30 min: *P<0.05 vs CP, †*P*<0.05 vs IP; AP 60 min: *P<0.05 vs CP, †*P*<0.05 vs IP; EDP 5 min: *P<0.02 vs CP; †*P*<0.0001 vs IP; EDP 30 min: *P<0.003 vs CP; †*P*<0.0001 vs IP; EDP 60 min: *P<0.003 vs CP, †*P*<0.003 vs IP.

**Table 3.** Parameters Relating to Ischemic Contracture

	N	T_0_, min	V_c_, mm Hg/min	A_max_, mm Hg	T_peak_, min	A_end_, mm Hg
CP	16	13.02±0.42	9.40±1.18	53.85±3.87	18.91±0.56	35.51±1.78
CP+Ac	12	12.87±0.36	13.6±2.0	62.73±3.73	17.72±0.58	37.30±1.81
CP+HG	12	13.78±0.30	21.41±2.74*	76.21±3.44*	17.14±0.49	45.93±1.77
CP+CaC	11	10.92±0.56*	10.00±1.51	56.91±3.40	16.37±0.77	35.41±2.75
IP	23	8.91±0.58*	7.78±0.67*	65.54±4.22	16.88±0.67	41.64±2.62
IP+Ac	13	6.49±0.72†	19.89±2.80†	102.03±7.48†	11.83±1.14	50.90±2.93
IP+HG	12	11.86±0.59†	11.54±1.32†	70.60±3.73	17.56±0.70	44.80±2.08

Hearts were perfused according to the protocols described in Figure 1. All the data presented in the table were obtained during the index ischemia. T_0_ indicates time at which rigor started; V_0_, average speed at which rigor reached the maximum amplitude; A_max_, rigor maximum amplitude; T_peak_, necessary time to reach A_max_; A_end_, rigor value at the end of the index ischemia; CP, control; Ac, sodium acetate; HG, high glucose; CaC, calcium challenge; IP, ischemic preconditioning.

T_0_: *P<0.005 vs CP, ^†^*P*<0.005 vs IP; V_0_: *P<0.03 vs CP, ^†^*P*<0.008 vs IP; A_max_, *P<0.001 vs CP, ^†^*P*<0.0001 vs IP.

### mtHK2 Dissociation Alone Does Not Increase OMM Permeabilization

**Figure 10.**
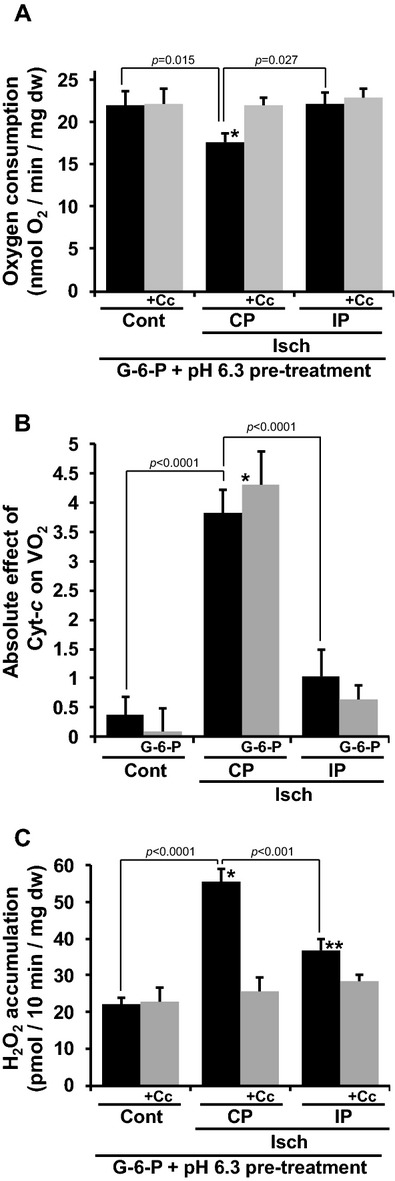
mtHK2 dissociation alone does not affect OMM permeabilization. Three groups of heart were studied as described in Figure 1. Fibers were permeabilized and washed twice in solution B at pH 6.3 (4°C) in presence of 10 mmol/L G‐6‐P as described in the Materials and Methods section. (A) Oxygen consumption of fibers in State 3 monitored in the absence (black bar) or presence of 12.5 lmol/L exogenous cytochrome c (gray bar, +Cc). (B) Absolute difference between rates of State 3 respiration of fibers measured in presence and absence of 12.5 lmol/L cytochrome c, respectively. Three groups of fibers were studied as described in (A). In each individual group, fibers were permeabilized and washed twice in a solution at pH 6.3 (4°C) in the absence (black bar) or presence of 10 mmol/L G‐6‐P (gray bar, G‐6‐P). (C) H2O2 accumulation monitored in the fibers described in (A), respiring in State 3 and monitored in the absence (black bar) or presence of 12.5 lmol/L exogenous cytochrome c (gray bar, +Cc). Data are presented as means±SEM. For each individual fiber preparations, the data from 2 fibers were averaged. (A) Cont (n=5), CP Isch (n=4), IP Isch (n=3), ANOVA P=0.0149, *P<0.05 vs CP Isch +Cc. (B) Cont (n=12), CP Isch (n=8), IP Isch (n=9), Cont+G‐6‐P (n=5), CP Isch+G‐6‐P (n=4), IP Isch+G‐6‐P (n=3), ANOVA P<0.0001 among all groups, *P<0.05 vs Cont+G‐6‐P. (C) Cont (n=5), CP Isch (n=5), IP Isch (n=3), ANOVA among groups without Cc P<0.0001, *P<0.05 vs CP Isch+Cc, **P=0.088 vs IP Isch+Cc. Cont indicates normoxic fibers, CP Isch: ischemic fibers, IP Isch: preconditioned ischemic fibers; CP, control; Ac, sodium acetate; HG, high glucose; CaC, calcium challenge; IP, ischemic preconditioning; mtHK2, mitochondria‐bound hexokinase 2; G‐6‐P, glucose‐6‐phosphate; OMM, outer mitochondrial membrane.

We hypothesized that mtHK2 might prevent the OMM permeabilization and cytochrome c release mediated by proapoptotic Bcl2 family members. To test this possibility, permeabilized fibers were prepared from normoxic control, end‐ischemic control, and IP‐end‐ischemic hearts and then subjected to incubation with G‐6‐P at pH 6.3 to dissociate the mtHK2. Rates of ADP‐stimulated respiration and H_2_O_2_ production were determined in the presence and absence of added cytochrome c and revealed that dissociation of mtHK2 was without significant effect on either parameter (Figures 10A and 10C compared with Figure 2C and 2D). The absolute effect of cytochrome c addition on the rate of respiration confirms this (Figure 10B).

### Rates of Phosphocreatine Production In Vitro and Ex Vivo Suggests IMM/OMM Contact Sites May Play a Role in the Inhibition of OMM Permeabilization by mtHK2

**Figure 11.**
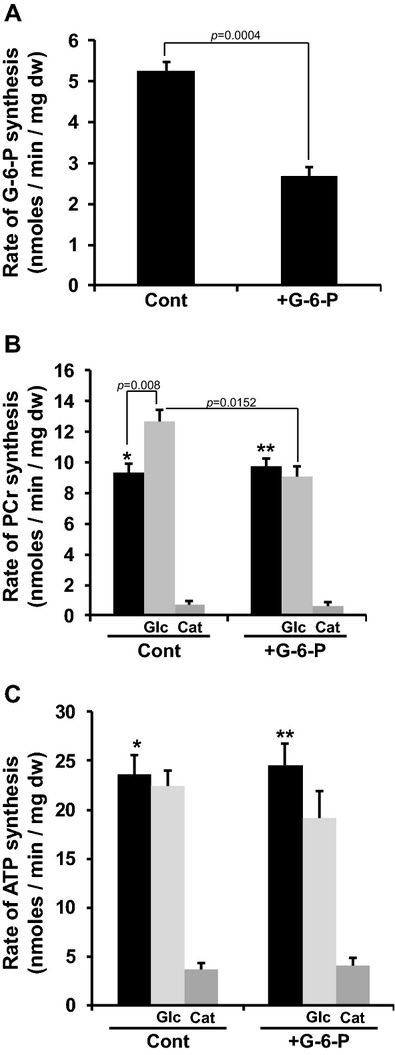
mtHK2 influences the rate of mitochondrial PCr synthesis in vitro. Normoxic control fibers were permeabilized and washed twice in solution B at pH 6.3 (4°C) in the absence (Cont) or presence of G‐6‐P 15 mmol/L (+G‐6‐P). Fibers were incubated with stirring in the presence of ADP 1 mmol/L, creatine 10 mmol/L, and, where indicated, glucose 5 mmol/L (Glc) for 5 minutes as described in Materials and Methods. In a separate group of experiments fibers were incubated as described earlier in a medium supplemented with carboxyatractyloside 5 μmol/L (Cat). (A) G‐6‐P output of permeabilized fibers. (B) Mitochondrial PCr output of permeabilized fibers. (C) Mitochondrial ATP output of permeabilized fibers. For each individual fiber preparation (ie, n number), the data from 2 fibers were averaged. (A) Cont (n=7) and Cont+G‐6‐P (n=9). (B and C) Cont no addition (n=7), Cont+Glc (n=3), Cont+Cat (n=3), +G‐6‐P no further addition (n=7), +G‐6‐P+Glc (n=3), and +G‐6‐P+Cat (n=3). (B) *P<0.05 vs Cont with Cat, **P<0.05 vs +G‐6‐P with Cat. (C) *P<0.05 vs Cont with Cat, **P<0.05 vs +G6P with Cat. mtHK2 indicates mitochondria‐bound hexokinase 2; G‐6‐P, glucose‐6‐phosphate; PCr, phosphocreatine.

MtHK2 is thought to play an important role in stabilizing mitochondrial contact sites,^29–30^ and dissociation of contact sites can enhance OMM permeabilization, cytochrome c release, and mPTP opening.^31–32^ To investigate whether mtHK2 dissociation might lead to greater disruption of mitochondrial contact sites, we took advantage of the role that they are believed to play in transporting ATP generated within the mitochondria to the cytosol as PCr.^32–33^ Thus, if ischemia leads to disruption of contact site as a result of mtHK2 loss, this should be reflected in a decreased rate of extramitochondrial PCr synthesis that would be ameliorated by IP. When normoxic control permeabilized fibers were depleted of mtHK by G‐6‐P incubation at pH 6.3, we detected a 50% decrease in the rate of G‐6‐P output (Figure 11A) as predicted, and this was accompanied by a significant (30%) decrease in the rate of PCr synthesis (Figure 11B). However, the rate of ATP production by the corresponding fibers was not significantly affected (Figure 11C).

**Figure 12.**
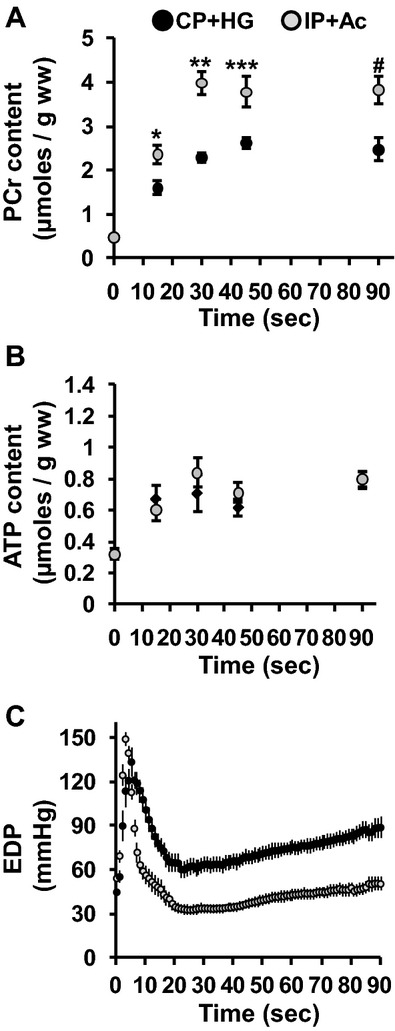
mtHK2 influences the rate of mitochondrial PCr synthesis ex vivo. Two groups of hearts were perfused according to the protocol described in Figure 1. (A and B) PCr and ATP content during the first 90 seconds of reperfusion measured in freeze‐clamped hearts characterized by either low or high end‐ischemic mtHK2 content (CP+HG and IP+Ac, respectively—see Figure 8). (C) End‐diastolic pressure (EDP) for the same hearts. Data are presented as means±SEM of 4 individual hearts per condition and time point. (A) *P=0.032 vs CP+HG 15 seconds, **P=0.012 vs CP+HG 30 seconds, ***P=0.039 vs CP+HG 45 seconds, ^#^*P*=0.0159 vs CP+HG 90 seconds. mtHK2 indicates mitochondria‐bound hexokinase 2; CP, control; Ac, sodium acetate; HG, high glucose; CaC, calcium challenge; IP, ischemic preconditioning; PCr, phosphocreatine.

**Figure 13.**
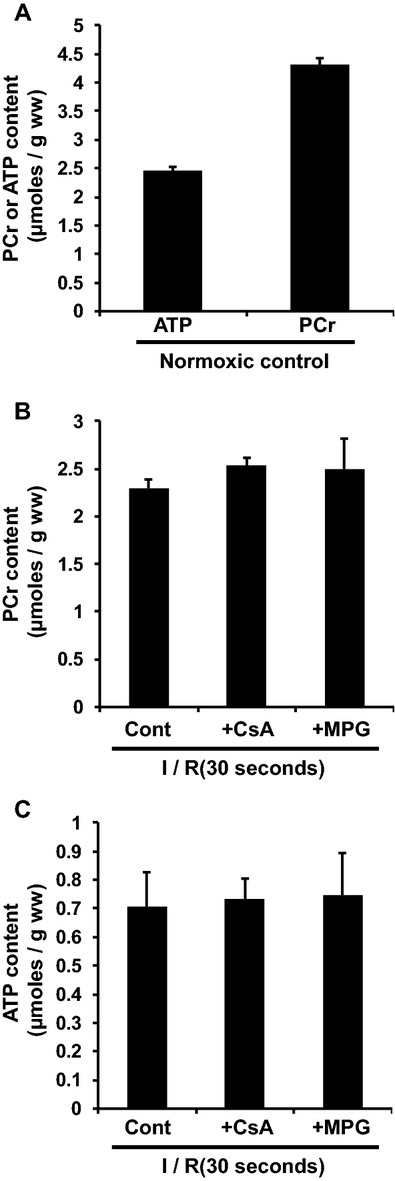
Effects of mPTP inhibition on heart bioenergetic status during early reperfusion after 30 minutes of ischemia. Hearts were perfused according to the protocols described in Figure 1 (see CP+HG group). After 30 minutes of ischemia and 30 seconds of reperfusion (I/R [30 seconds]), hearts were freeze‐clamped and then grounded in liquid nitrogen. Phosphocreatine (PCr) and ATP were determined by enzymatic assay as described in Materials and Methods. (A) Content of ATP and PCr obtained in a normoxic control group (n=6). (B) PCr content in the group CP+HG (Cont) treated for 15 minutes before ischemia and during 30 seconds of reperfuson with cyclosporin A 0.2 μmol/L (+CsA) or *N*‐(2‐mercaptopropionylglycine) 2 mmol/L (+MPG). (B and C) n=4 in each group. mPTP indicates mitochondrial permeability transition pore; CP, control; HG, high glucose.

To investigate the effects of ischemia and IP on the stability of contact sites in situ, we freeze‐clamped hearts at various time points during the first 90 seconds of reperfusion after 30 minutes of ischemia and determined their PCr and ATP contents. We used hearts perfused with high glucose and IP hearts perfused with acetate because these demonstrated the lowest and highest mtHK2, respectively, as shown in Figure 9. The data of Figure 12 show that after 15 seconds of reperfusion, the content of PCr (Figure 12A) but not of ATP (Figure 12B) of IP hearts perfused with acetate (highest mtHK2) was significantly greater than in control hearts perfused with glucose hearts (lowest mtHK2), and this difference was maintained throughout the 90 seconds studied. Moreover, the higher PCr content was accompanied by less end‐diastolic dysfunction during the onset of reperfusion (Figure 12C). These data are consistent with greater contact site stabilization in the IP hearts perfused with acetate leading to enhanced rates of PCr synthesis in the cytosol. This would enable faster reuptake into the sarcoplasmic reticulum of the calcium that accumulates during ischemia and so a more rapid decline in the EDP. We confirmed that the PCr deficit in the control hearts perfused with glucose group was unlikely to be a result of mPTP opening because preischemic treatment of hearts with an mPTP inhibitor (0.2 μmol/L CsA) or ROS scavenger [2 mmol/L *N*‐(2‐mercaptopropionylglycine)[ was without effect on PCr and ATP levels after 30 seconds of reperfusion (Figure 13).

## Discussion

In this article, we address the mechanisms by which IP modulates mitochondrial function during ischemia so as to prevent mPTP opening during reperfusion and mediate cardioprotection. Our data suggest that prevention of mtHK2 dissociation, contact site disruption, and OMM permeabilization during ischemia play important roles.

### Bcl2 Family Members and OMM Permeabilization During Ischemia

We have previously suggested that the decrease in mitochondrial Bcl‐x_L_ content observed at the end of ischemia could be responsible for cytochrome c release.^8^ Loss of this antiapoptotic protein could unmask the activity of proapoptotic proteins such as Bax and Bak whose genetic deletion provides powerful protection against reperfusion injury.^12^ However, here we show that although IP prevented the increased permeabilization of the OMM to cytochrome c (Figure 2), it did not prevent the decrease in Bcl‐x_L_ (Figure 3A and 3B). Nor could we observe an effect of ischemia or IP on the recruitment of activated Bax to mitochondria (Figure 3C through 3F). Thus, changes in Bcl2 family members alone cannot account for the prevention of OMM permeabilization at the end of ischemia by IP. This implies that other factors are masking the permeability pathway for cytochrome c, and our data strongly suggest that mtHK2 may play such a role.

### Extent of Reperfusion Injury Correlates With Loss of mtHK2 During Ischemia

**Figure 14.**
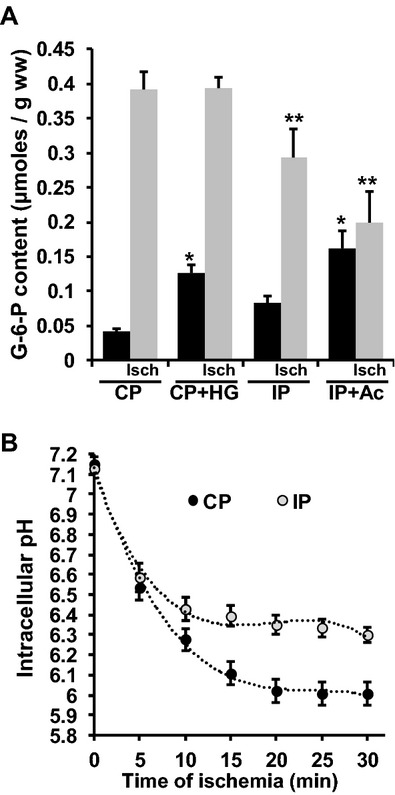
Effects of ischemia on G‐6‐P accumulation and intracellular pH. (A) Hearts were perfused according to the protocol described in Figure 1. Hearts were then freeze‐clamped before ischemia or after 10 minutes of global ischemia (Isch) and their content in G‐6‐P was assessed as described in Materials and Methods. Data are presented as means±SEM, n=4 for each group. (A) ANOVA *P*=0.0009 among preischemic groups and *P*=0.0026 among ischemic groups, *P<0.05 vs CP, **P<0.05 vs CP Isch. (B) Data represent the evolution of the Langendorff‐perfused rat heart intracellular pH during global ischemia monitored by ^31^P NMR. Data were extracted from the following publications^27,36–42^ and plotted using Microsoft Excel. G‐6‐P indicates glucose‐6‐phosphate; CP, control; Ac, sodium acetate; HG, high glucose; CaC, calcium challenge; IP, ischemic preconditioning.

Ischemia has been shown to cause a loss of mtHK2^8,11^ that is prevented by IP.^11^ We confirm this here (Figure 4) and explored the relationship between mtHK2 binding and ischemia–reperfusion injury in more detail. Using permeabilized fibers, we showed that mtHK2 could be dissociated from mitochondria by the combination of low pH (6.3) and high G‐6‐P (Figure 5B). IP has been shown to decrease G‐6‐P accumulation,^34–35^ as we confirm in Figure 14A, and many workers have reported less acidification during ischemia after IP (Figure 14B). By applying other preischemic perfusion protocols that stimulated or inhibited glycolysis and glycogen breakdown,^27^ we generated 7 groups of hearts whose mitochondria displayed a range of hexokinase activities at the end of ischemia from 30 to 90 mU/mg protein (Figure 8A). Importantly, when mtHK enzymic activity was plotted against infarct size, there was a strong inverse correlation (Figure 9A) that Western blotting revealed was due to changes in mtHK2 (Figure 8B and 8C). These data provide further evidence for the importance of mtHK2 in cardioprotection and are consistent with mtHK2 acting to inhibit OMM permeabilization, cytochrome c release, and ROS production. This would lead to inhibition of mPTP opening on reperfusion. However, it is important to note that our data could also be explained by a direct inhibitory effect of mtHK2 on mPTP opening, which has been proposed by others.^13–14^

### Mechanism of mtHK2 Dissociation From Mitochondria in Ischemia

Permeabilized fibers provide an ideal system in which to study the mechanism and regulation of mtHK2 dissociation from mitochondria that may account for its loss during ischemia and the modulation of this by IP and other interventions. Although the release of mtHK2 from isolated mitochondria incubated with G‐6‐P has been described by others,^26^ we were unable to achieve this in permeabilized fibers unless we also reduced the pH to <7 (Figure 5). The optimal pH for dissociation was found to be 6.3 at 4°C with less dissociation at more acid pH values (Figure 5B). The bell‐shaped dissociation curve suggests the involvement of 2 ionizable groups in the binding of mtHK2 to mitochondria. One of these may be the histidine (pKa 6.0) that is present in the center of the hydrophobic N‐terminal peptide (MIASHMIACL in rat from accession number P27881) required for mitochondrial binding.^15^ It is likely that this histidine needs to be uncharged to insert into the OMM, and this would be its predominant ionization state at physiological pH. As the pH falls to <7, histidine becomes more charged, favoring dissociation of HK2. The other group involved may be the phosphate of G‐6‐P whose pKa is 6.11. If G‐6‐P must be deprotonated to bind to HK2, then as the pH drops to <6.1, its binding to HK2 would diminish, preventing the conformational change required for HK2 dissociation from the OMM. Together, these effects could generate the G‐6‐P concentration and pH dependence observed. Although HK2 is the major HK isoform in the heart, there is some HK1,^43^ but this does not dissociate during ischemia,^8,11^ perhaps because it does not have a histidine in the equivalent N‐terminal sequence (MIAAQLLAYY in rat from accession number P05708).

**Figure 15.**
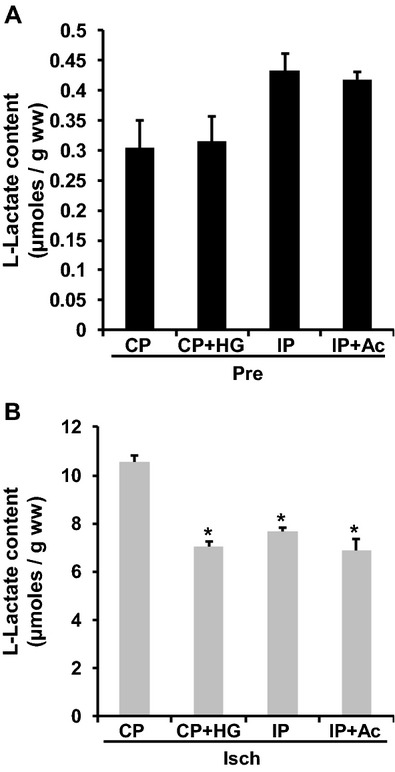
Effects of ischemia on l‐lactate accumulation. Four groups of hearts were perfused according to the protocol described in Figure 1. Hearts were then freeze‐clamped prior to ischemia (A) or after 10 minutes of global ischemia (B) and their content in l‐lactate was determined as described in the Materials and Methods. Data are presented as means±SEM, n=4 for each group of hearts. (B) ANOVA among ischemic groups *P*<0.0001, *P<0.05 vs CP Isch. CP indicates control; Ac, sodium acetate; HG, high glucose; CaC, calcium challenge; IP, ischemic preconditioning.

To provide some indication as to whether the major cause of HK2 dissociation during ischemia was G‐6‐P or low pH, G‐6‐P content was determined in hearts at the end of ischemia in 4 groups of hearts showing a wide range of mtHK2—control, control high glucose, IP, and IP plus acetate (Figure 14A). The l‐lactate content was also determined (Figure 15) as an indicator of lactic acid production during ischemia and showed a similar pattern to the rates of glycogen breakdown determined from the glycogen content before and after ischemia (Figure 6A and 6B). These data are consistent with mtHK2 dissociation during ischemia being mediated by both the drop in pH and the rise in G‐6‐P, with cardioprotective protocols such as IP modulating either or both of these parameters. However, this does not rule out additional mechanisms such as phosphorylation of the voltage dependent anion channel (VDAC) via the different protein kinase pathways implicated in IP,^14–15^ although some of these such as the Akt/glycogen synthase kinase‐3beta (GSK3 beta) pathway could exert their effects indirectly by enhancing glycogen breakdown.

### Role of mtHK2 Dissociation in OMM Permeabilization and Cytochrome c Release

Although mtHK2 dissociation has been reported by others to favor cytochrome c release.^26^ our data with permeabilized fibers isolated from ischemic hearts provide no evidence for this. Thus, dissociation of mtHK2 via incubation with G‐6‐P at pH 6.3 was without effect on cytochrome c stimulation of either state 3 respiration or H_2_O_2_ production (Figure 10). These data demonstrate that dissociation of mtHK2 alone does not induce OMM permeabilization and imply that additional factors must be required. Activation of proapoptotic members of the Bcl2 family is an obvious possibility because ischemia is associated with the loss of Bcl‐x_L_ (Figures 3A and 3B). However, after ischemia, IP hearts show as much loss of Bcl‐x_L_ as control hearts, yet treatment of fibers with G‐6‐P at pH 6.3 to dissociate mtHK2 was still without effect on cytochrome c stimulation of either state 3 respiration or H_2_O_2_ production (Figure 10). These data imply that yet another mechanism must play a critical role in OMM permeabilization, and our data suggest that this may be the disruption of contact sites between the IMM and OMM during ischemia.

### Disruption of Mitochondrial Contact Sites May Be Critical for OMM Permeabilization

Mitochondrial HK2 has been shown to associate with contact sites that link the IMM and OMM. Contact sites are thought to contain several proteins including VDAC1 and the peripheral benzodiazepine receptor (also known as translocator protein) of the OMM and ANT of the IMM. They may also represent the binding site of members of the Bcl2 family.^32^ Their disruption is thought to enhance the permeability of the OMM to cytochrome c and to increase the sensitivity of the mPTP to [Ca^2+^].^31–32^ Indeed, this may explain how ligands of the translocator protein can inhibit mPTP opening^44^ and mediate protection from ischemia–reperfusion injury.^45^ Importantly, contact sites can be disrupted by elevated matrix [Ca^2+^],^31,46^ and the extent of [Ca^2+^] increase at the end of ischemia has been shown to be a good indicator of subsequent cell death in an isolated myocyte model of ischemia–reperfusion.^47^ Furthermore, there is evidence that contact sites are decreased after ischemia^33^ and that hearts from mice deficient in mitochondrial creatine kinase are more sensitive to ischemia–reperfusion injury.^48^

A key role of contact sites in the normal heart is thought to be the efficient transport of mitochondrial ATP to the cytosol as PCr.^32–33^ Indeed, there is good evidence for an impairment of the PCr shuttle after ischemia,^49^ which is improved after IP.^50^ Thus, if mtHK2 is involved in stabilizing the contact sites, its dissociation should be reflected in a decreased rate of PCr output from the mitochondria. This is what we observed after preincubation of permeabilized fibers with G‐6‐P at pH 6.3, although the effect was only observed when glucose was also present to enable HK2 activity (Figure 11B). The reason for this glucose requirement is not known, but others have reported that the protective effect of mtHK binding on cell survival also requires the presence of glucose.^51^ To assess the extent of contact site disruption at the end of 30 minutes of ischemia in situ and its relationship to mtHK2 binding, we used initial rates of PCr synthesis in the first phase (15 to 90 seconds) of reperfusion. As predicted, we observed that the control plus high‐glucose hearts (greatest loss of mtHK2 and infarct size) displayed significantly slower rates of PCr recovery on reperfusion than did the IP plus acetate hearts (highest mtHK2 and smallest infarct size) (Figures 9A and 12A). This effect is unlikely to be secondary to mPTP opening because the deoxyglucose entrapment technique shows mPTP opening does not occur in the first 2 minutes of reperfusion when pH_i_ remains at pH <7.^10^ Furthermore, we observed no differences in the ATP content between these 2 groups of hearts. Nor did pretreatment with CsA 0.2 μmol/L or MPG 2 mmol/L for 15 minutes before ischemia and during reperfusion (to inhibit mPTP opening) have any effect on PCr or ATP levels. Thus, our data are entirely consistent with the hypothesis that maintaining mtHK2 binding during ischemia reduces contact site disruption.

## Conclusions

The data we present in this article support the hypothesis that a critical factor in the determining the extent of damage (infarct size) during reperfusion after ischemia is loss of HK2 bound to mitochondria. This is entirely consistent with the demonstration that hearts from heterozygous HK2 knockout mice are more sensitive to ischemia–reperfusion injury.^52^ We propose that this, together with increased [Ca^2+^], destabilizes contact sites during ischemia and enables cytochrome c permeation across the OMM. This is mediated by channels formed by unmasking proapoptotic members of the Bcl2 family such as Bax and Bak already in the OMM. We suggest that this unmasking is caused by a loss of Bcl‐x_L_ which might be mediated by proteolytic degradation involving caspases or calpains, inhibitors of which have been shown to be cardioprotective.^53^ Cytochrome c loss will lead to greater ROS production^8^, at least in part accounting for the oxidative stress observed after ischemia–reperfusion that results in mPTP opening.^1^ The loss of mtHK2 will also lead to inhibition of ATP channeling from the mitochondria to the cytoplasm and thus impaired reuptake of Ca^2+^ into the sarcoplasmic reticulum, as has been observed in creatine kinase–deficient mice.^48^ This, in turn, will lead to a greater uptake of Ca^2+^ into the mitochondria, which, in conjunction with the oxidative stress, will induce mPTP opening.^1^ By preventing mtHK2 dissociation, IP will maintain contact sites and reduce OMM permeabilization, leading to less oxidative stress and mitochondrial calcium overload and thus less mPTP opening and necrotic damage (infarct). Stabilizing contact sites may also inhibit mPTP opening directly,^14,31^ and this may provide an additional mechanism by which mtHK2 dissociation sensitizes the mPTP to [Ca^2+^].^13,44^ It should also be noted that mPTP opening itself induces cytochrome c loss by OMM rupture and increased ROS production, potentially leading to a cascading opening of the mPTP in adjacent mitochondria as reperfusion continues.^54–55^

**Figure 16.**
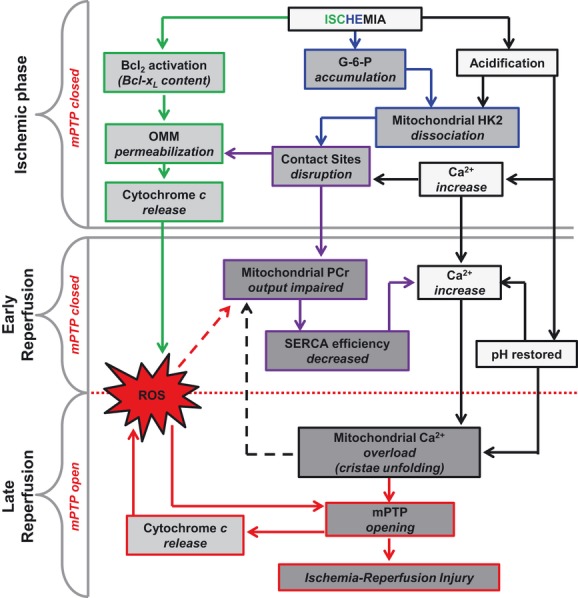
Scheme illustrating how mtHK2 dissociation might influence mPTP opening during reperfusion. During ischemia glucose catabolism leads to accumulation of G‐6‐P and H^+^ that causes mtHK2 dissociation. This, in combination with increased cytosolic [Ca^2+^] may induce mitochondrial contact site (MiCS) disruption. In parallel Bcl‐x_L_ content decreases and together these factors induce OMM permeabilization and cytochrome c release. During early reperfusion, restoration of physiological pH is accompanied by cytosolic and mitochondrial Ca^2+^ overload. Mitochondrial PCr output is impaired as a result of mtHK2 dissociation and MiCS disruption. This deficit in PCr may impair Ca^2+^ reuptake by the sarcoplasmic reticulum, leading to a more pronounced cytosolic and mitochondrial Ca^2+^ overload. The latter, in combination with the ROS produced during reperfusion as a result of cytochrome c release, would greatly favor mPTP opening and consequently lead to the development of ischemia–reperfusion injury. mtHK2 indicates mitochondria‐bound hexokinase 2; mPTP, permeability transition pore; OMM, outer mitochondrial membrane; ROS, reactive oxygen species; PCr, phosphocreatine. SERCA, sarcoendoplasmic reticulum calcium transport ATPase.

The hypothesis described here is summarized in Figure 16. An attractive feature of this proposal is that it has the potential to explain how a diverse range of known cardioprotective regimes and signaling pathways might produce the same final outcome of stabilizing contact sites, reducing OMM permeabilization, and inhibiting mPTP opening. For example, metabolic interventions might modulate mtHK2 binding through changes in G‐6‐P levels and pH_i_ secondary to alterations in glycogen metabolism. The latter can be regulated by a variety of kinase cascades including those implicated in IP such as PKC, Akt, and GSK3β pathways.^2–3^ Such kinases might also influence mtHK2 binding through other means including phosphorylation of OMM proteins such as VDAC.^56–57^ Another target for regulation is members of the Bcl2 family whose activity can also be regulated by phosphorylation, as well as proteolysis and translocation.^58^ Although the proposals of Figure 16 are appealing, we recognize that the methods we have used to study mitochondrial contact sites and their role in OMM permeabilization and mPTP opening are indirect. To test our hypothesis further, it will be necessary to find more direct ways of determining mitochondrial contact sites in situ.

## Acknowledgment

The authors would like to thank Léo Gerville‐Reache for his major contribution in the statistical analysis.

## Author Contribution

Philippe Pasdois and Andrew Halestrap devised and supervised the project. All the experiments were designed and performed by Philippe Pasdois except the Western blot studies which were carried out by Joanne E. Parker. The manuscript was written by Philippe Pasdois and Andrew Halestrap.
